# Supplementary material for: An Investigation on the Use of Au@SiO2@Au Nanomatryoshkas as Gap-Enhanced Raman Tags
Source: Nanomaterials (Basel). 2023 Nov 1;13(21):2893. doi: 10.3390/nano13212893 (PMC10650036; doi:10.3390/nano13212893)
Supplement: Supplementary file 1 [file nanomaterials-13-02893-s001.zip › nanomaterials-2662262-supplementary.pdf]

AN INVESTIGATION ON THE USE OF AU@SIO$_2$@AU NANOMATRYOSHKAS AS GAP ENHANCED RAMAN TAGS

SUPPORTING INFORMATION

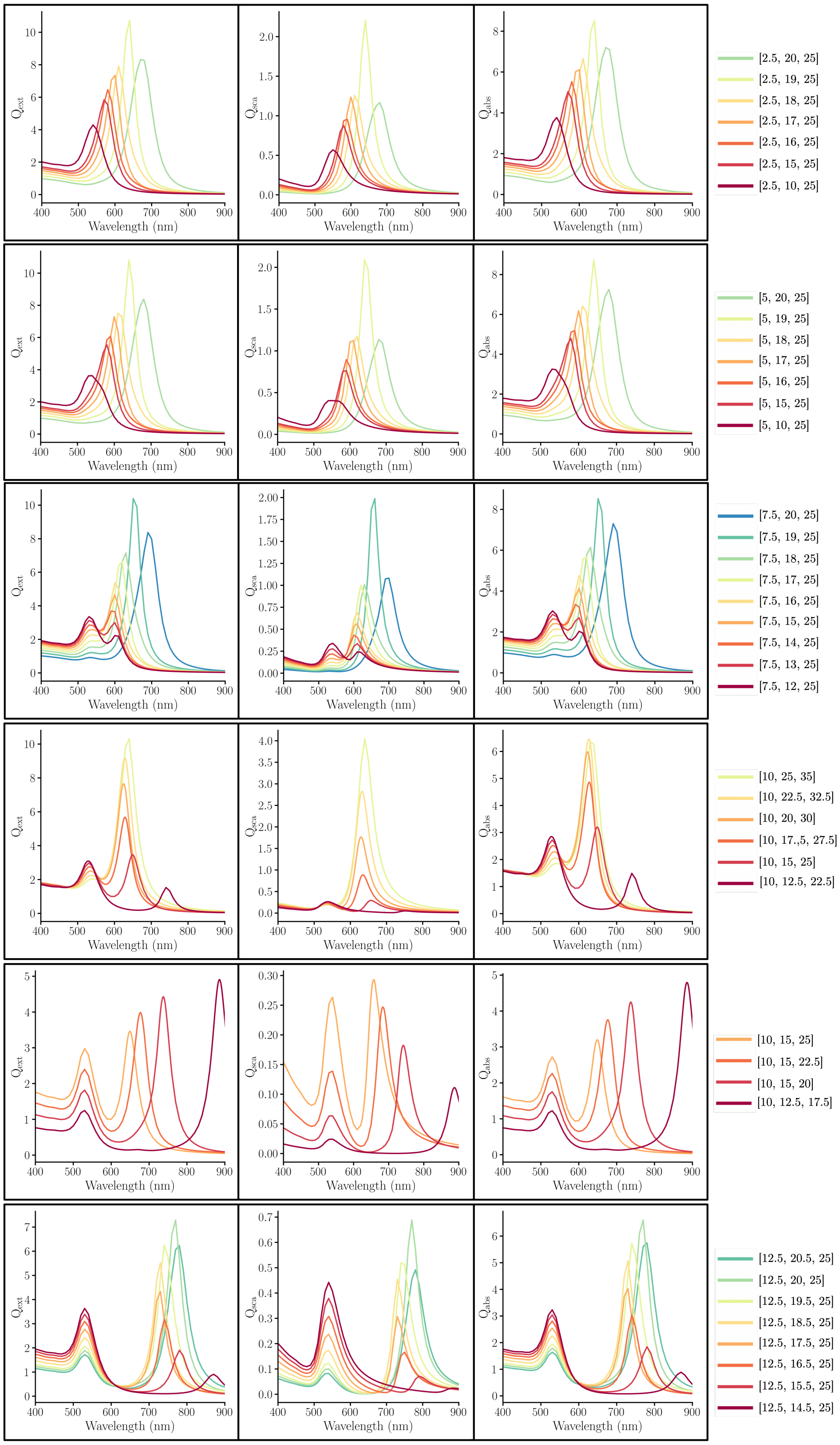

Figure S1: Q_{ext} (column 1), Q_{sca} (column 2), and Q_{abs} (column 3) spectra of nanomatryoshkas with different geometries using the following notation: [r_1, r_2, r_3] where r_1 is the radius of the Au core, r_2 is the radius of the SiO_2-coated Au core, and r_3 is the radius of the NM.

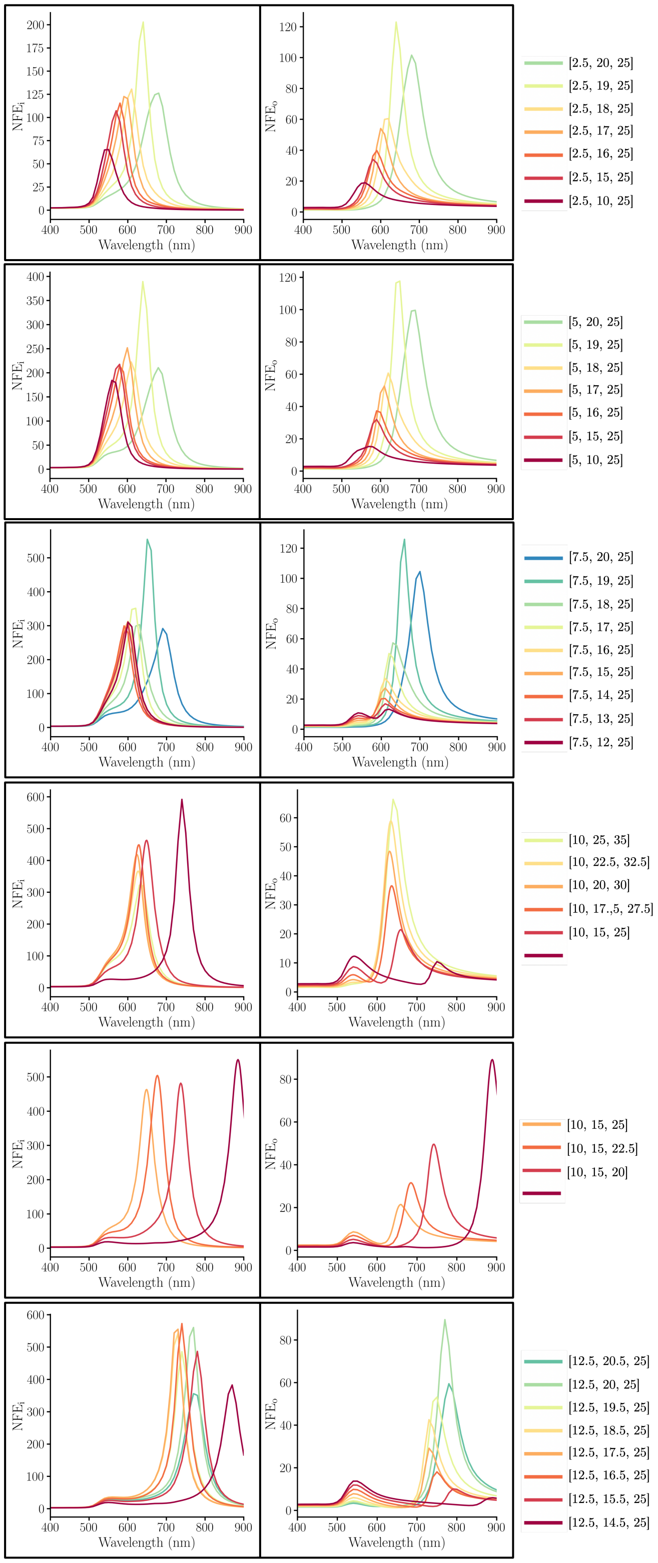

Figure S2: NFE$_i$ (column 1) and NFE$_o$ (column 2) spectra of nanomatryoshkas with different geometries using the following notation: [r_1, r_2, r_3] where r_1 is the radius of the Au core, r_2 is the radius of the SiO_2-coated Au core, and r_3 is the radius of the NM.

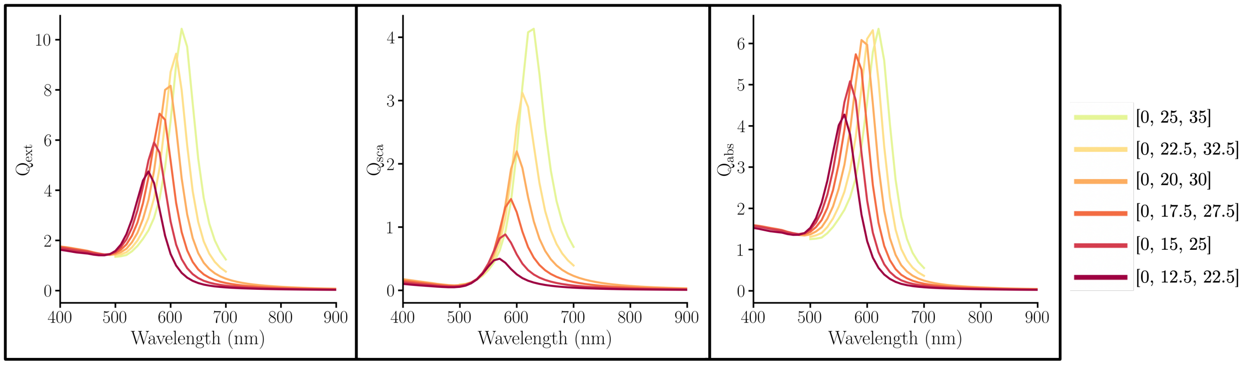

Figure S3: Q_{ext} (column 1), Q_{sca} (column 2), and Q_{abs} (column 3) spectra of nanoshells with different geometries using the following notation: [r_1, r_2, r_3] where r_1 is the radius of the Au core (absent here), r_2 is the radius of the SiO_2-coated Au core, and r_3 is the radius of the NM.

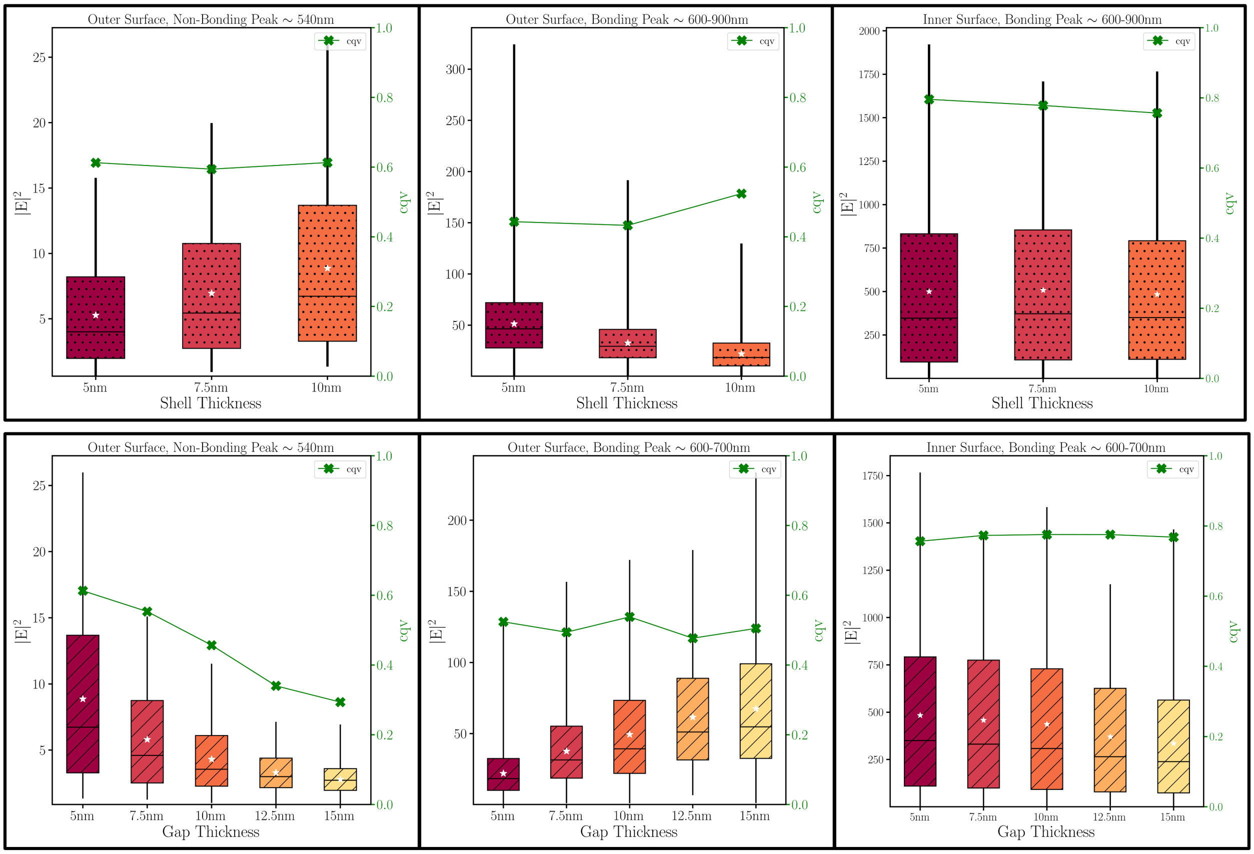

Figure S4: Box plots of the distribution of $|\boldsymbol{E}|^2$ intensities at 1nm above the surface of the shell for the changing gap series (first row) and the changing shell series (bottom row). White star represented the reported mean shown in NFE_o calculations. Green cross and line between represent cqv and trend.

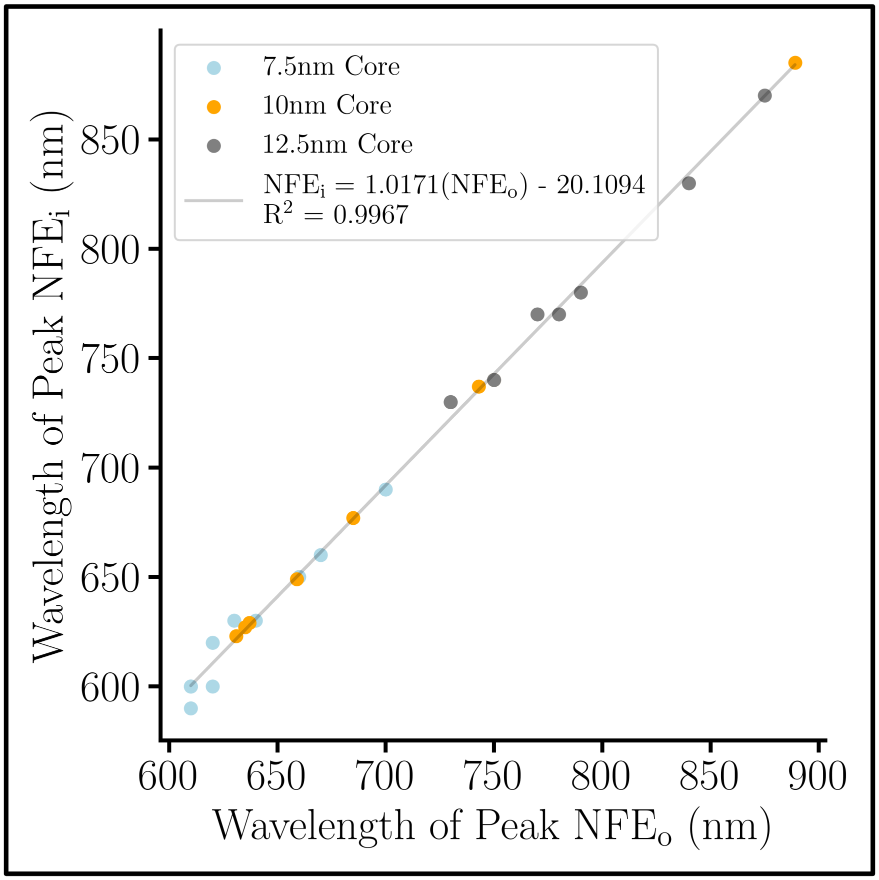

Figure S5: Correlation plot between NFE_i and NFE_o bonding peaks. For each color, a different series of systems were computed, each with a different radius for the core: 7.5nm (blue), 10nm (orange), and 12.5nm (grey). On average, the NFE_o bonding peak is redshifted 8.2nm from the NFE_i bonding peak.
